# The Impact of “Harmful Behaviours” on Parents of Autistic Children

**DOI:** 10.1177/13623613261476379

**Published:** 2026-08-19

**Authors:** Melanie Heyworth, Diana Weiting Tan, Elizabeth Pellicano

**Affiliations:** 1 Macquarie University, Sydney, New South Wales, Australia; 2 Reframing Autism, Sydney, New South Wales, Australia; 3 La Trobe University, Melbourne, Victoria, Australia; 4 University of Western Australia, Perth, Australia; 5 University College London, UK

**Keywords:** autism, parents, harmful behaviours, externalising behaviours, trauma

## Abstract

In existing literature, an Autistic child’s “externalising behaviour” (e.g., aggression, defiance) is often associated with poor parental mental health outcomes. Yet few studies have considered the extent and nature of the impact of a child’s harmful behaviours (physical harm to self, others, and property) on parents qualitatively. Here, we examined how Autistic and non-autistic parents conceptualise their Autistic children’s harmful behaviours, and the impact of such behaviours on parents. We analysed 39 Autistic and non-autistic parent interviews using reflexive thematic analysis, from which we identified four themes. Theme 1 encompassed parents’ experiences of their children’s harmful behaviours. Theme 2 captured parents’ explanations for their children’s harmful behaviours, which they understood as indicating intense distress. In Theme 3, parents reflected on their responses to periods of crisis, during which they prioritised safety but acknowledged a systemic lack of support hindered their efforts. Finally, Theme 4 describes the detrimental impacts on parent mental health, including feelings of helplessness, grief, guilt, and fear for the future. We show that it is imperative to more openly discuss child harmful behaviours in neuroaffirming ways, to develop responsive and appropriate parental and familial supports, which acknowledge the role of guilt and trauma in parental mental health.

**Lay Abstract:**

Lots of research suggests that an Autistic child’s so-called “externalising behaviour” (like aggression or defiance) has a negative impact on that child’s parents’ mental health. But, so far, very few studies have talked directly to parents about why and how they experience distress because of their child’s externalising behaviours. In this study, we interviewed 39 Autistic and non-Autistic parents of Autistic children, to better understand their experiences of specific externalising behaviours. For this study, we were particularly interested in children’s “harmful behaviours,” which we defined as violence towards self, others, and/or property. We wanted parents to tell us in their own words what their experiences of such harmful behaviours were, how they understood these periods of crisis, what they did to respond to their children’s harmful behaviours, and what they felt about the behaviours. When we analysed parents’ responses, we came up with four main ideas or “themes.” Parents saw harmful behaviours as a combination of child self-harm, suicidal behaviour, and physical violence to their parents, siblings, and property (Theme 1). But parents did not necessarily blame their child, instead understanding that their child’s behaviour was likely an indication of intense or extreme distress or dysregulation (Theme 2). Parents wanted to keep their whole family safe, but they found they had limited options to do this, and they felt there was little or no support to help them during crises (Theme 3). Unfortunately, parents expressed lots of different emotions, like helplessness, grief, guilt, and fear, as a result of their child’s harmful behaviours (Theme 4). We need to understand better what is happening in families, how they are responding, and what support they need to cope so that we can help families who are experiencing these kinds of crises. This article addresses these questions.

## Introduction

Parents of Autistic^1^ children report poorer well-being outcomes than both parents of typically developing children and children with other disabilities (Cai et al., 2020), manifested as increased stress and depression (Enea & Rusu, 2020; Lam et al., 2024), and decreased individual and familial quality of life (e.g., Hsiao, 2018; Temelturk et al., 2021; Vasilopoulou & Nisbet, 2016). The reasons for such outcomes are varied and complex, although the level of an Autistic child’s so-called “externalising behaviour” is often cited (e.g., Mello et al., 2022; Olson et al., 2022; Yorke et al., 2018). “Externalising behaviours” include those that risk “‘the physical safety of the person or others’ . . . [and] that others may find challenging (e.g., aggression, non-compliance, tantrums, property damage)” (Suvarna et al., 2024, p. 236).

Many quantitative studies have reported correlations between Autistic children’s “externalising behaviours” and parental mental health outcomes (Barroso et al., 2017; Paynter et al., 2024; Suvarna et al., 2024; Yorke et al., 2018). However, few studies have sought to understand in-depth parents’ experiences of their Autistic children’s “externalising behaviours.” The few existing qualitative studies focus on specific domains of harm, such as child to parent violence (Holt, 2023, 2024; Swaab et al., 2017, 2023).

Such reticence to explore parents’ experiences of their Autistic child’s “externalising behaviours” qualitatively may be partly underpinned by a concern to avoid parent-blaming. For many, Bettelheim’s “refrigerator mother” hypothesis, which attributed a child’s autism to disordered maternal attachment, has a continued influence (Heyworth, 2024), which has made it difficult to examine parenting Autistic children in constructive ways (Briggs, 2020; van Rosmalen et al., 2020; Ventola et al., 2017). As Moll and Krishnan (2025) argue, until recently, discussing parenting experiences in the context of a child’s neurodevelopmental conditions has been a “taboo” topic given the sensitivity of the “bad mother” inheritance. When coupled with the stigma that parenting an Autistic child who displays externalising behaviours often brings (Swaab et al., 2023, 2026), it is perhaps unsurprising that researchers have shied away from such a confronting and delicate subject (Holt, 2024).

Such reticence may also stem from a move towards neurodiversity-affirming research, which eschews pathologising autism and seeks to situate Autistic challenges outside of the individual and within their social and environmental context (Pellicano & den Houting, 2022). Undoubtedly, discussing Autistic children’s harmful “externalising behaviours” is challenging to do in ways that respect the child and their parent, reflect the complexity of their individual and collective experiences, and avoid deficits-based descriptions of the behaviour itself. For neurodiversity-affirming researchers, the impetus to avoid *child*-blaming – and, indeed, *autism-*blaming – is as vital as it is to avoid parent-blaming. Moreover, the term “externalising behaviours” can be used as a “catchall” to include behaviours that are not inherently harmful (e.g., stimming), undermining neurodiversity-affirming approaches by potentially stigmatising intrinsically Autistic behaviours. For this reason, we adopt the term “harmful behaviours” in this article to better capture the risk of harm to self, others, and property.

Researchers’ apparent reluctance to explore the impact of Autistic children’s harmful behaviours is understandable. Yet, in so doing, we have failed to find ways to speak respectfully about the tangible, detrimental, and often desperate situation facing families who are living daily with child harmful behaviours (Stewart et al., 2017) – and we have thus failed to acknowledge the very real and significant needs of these families. This study aims to redress this imbalance.

The current data are drawn from a broader study, in which 80 parents reflected on their experiences parenting Autistic children (see Heyworth et al., 2025). A subset of these parents spoke spontaneously about experiences of child violence (towards self, others or property), which were often seen as “crisis” points for the child, the parent, and their family. Here, we analyse this subset of responses, thus asking: How do parents conceptualise their Autistic children’s physically harmful behaviours, and in what ways do such behaviours impact parents’ self-reported mental well-being?

## Method

### Participants

Using both convenience and snowball sampling utilising M.H.’s community and social media networks, 80 parents of Autistic children were recruited for a study examining experiences of parenting Autistic children. Eligibility criteria included being a parent of at least one Autistic child aged 6 to 12 years with a clinical autism diagnosis, residing in Australia, and being comfortable to communicate in English.

Of these 80 parents, 39 (48.7%) spoke spontaneously about their Autistic child’s physically harmful behaviours. Participants who spoke (even briefly) about their child’s physically harmful behaviours were included in this analysis to better understand the nature and impact of such behaviours. Of the 39 participants included here, 19 were Autistic (n = 13 [68.4%] formally diagnosed and n = 6 [31.6%] self-identified), and 20 (51.3%) were non-Autistic. Thirty-four (87.2%) identified as women and 33 (84.6%) were born in Australia (see Tables 1 and 2).

**Table 1.** Participant Characteristics.

Participant characteristics	Parents of Autistic children (*n* = 39)		
	All parents (*n* = 39)	Autistic parents (*n* = 19)^a^	Non-autistic parents (*n* = 20)^b^
	*M* (*SD*), range, or *N* (%)^c^		
Age (years)	42.6 (4.9) 31.9–51.5	42.2 (4.9) 31.9–51.5	43 (5) 35.3–50.6
Age at autism diagnosis (years)^d^	NA	31–50 *M* = 39.2, *SD* = 5	NA
Interview duration (min)	91.7 (25) 43–195	83.2 (18.8)^e^ 43–120	100.1 (24.9) 70–195
Gender			
Woman	34 (87.2%)	15 (78.9%)	19 (95%)
Man	3 (7.7%)	2 (10.5%)	1 (5%)
Nonbinary	2 (5.1%)	2 (10.5%)	0 (0%)
Country of birth			
Australia	33 (84.6%)	15 (78.9%)	18 (90%)
Outside Australia	6 (15.4%)	4 (21.1%)	2 (10%)
Parenting arrangements			
Co-parenting	15 (38.5%)	8 (42.1%)	7 (35%)
Primary caregiver	16 (41%)	6 (31.6%)	10 (50%)
Solo parenting	8 (20.5%)	5 (26.3%)	3 (15%)
Educational attainment			
High school	3 (7.7%)	2 (10.5%)	1 (5%)
Post-school qualification	35 (89.8%)	16 (84.2%)	19 (95%)
Other	1 (2.6%)	1 (5.3%)	0 (0%)
Co-occurring conditions			
Attention-deficit hyperactivity disorder (ADHD)	15 (38.5%)	12 (63.2%)	3 (15%)
Anxiety	19 (48.7%)	11 (57.9%)	8 (40%)
Depression	15 (38.5%)	9 (47.4%)	6 (30%)
Post-traumatic stress disorder (PTSD)	10 (25.6%)	7 (36.8%)	3 (15%)

a Two participants identified as “questioning” their Autistic identity in the demographic survey; since they self-identified as Autistic in the interview, they have been coded as Autistic for the purposes of this analysis. ^b^Two participants identified as “questioning” their Autistic identity in the demographic survey; since they did not self-identify as Autistic in the interview, they have been coded as non-autistic for the purposes of this analysis. ^c^Data are mean (*SD*; range) or *n* (%). Percentages may not sum to 100% due to rounding issues. NA = not applicable. ^d^Age at diagnosis, or age at which participants began to self-identify (in years). ^e^One participant chose to complete their interview in writing.

**Table 2.** Autistic Child Characteristics, as Reported by Parents.

Child characteristics	All parent participants (*n* = 39)	Autistic parent participants (*n* = 19)	Non-autistic parent participants (*n* = 20)
	*M* (*SD*), range, or *N* (%)^a^		
Total number of Autistic children	*n* = 39	*n* = 19	*n* = 20
Age (years)	10.4 (1.8) 6.3–12.9	10.7 (1.7) 7.8–12.8	10 (1.8) 6.3–12.9
Age at autism diagnosis (years)	6.9 (2.7) 1.1–11.75	6.9 (2.9) 1.1–11	6.39 (2.6) 3–11.75
Gender			
Girl	15 (38.5%)	8 (42.1%)	7 (35%)
Boy	23 (59%)	10 (52.6%)	13 (65%)
Nonbinary	1 (2.6%)	1 (5.3%)	0 (0%)
Communication			
Speaking	37 (94.9%)	18 (94.7%)	19 (95%)
Nonspeaking	2 (5.1%)	1 (5.3%)	1 (5%)
Current educational setting			
Mainstream (with or without support)	26 (66.7%)	11 (57.9%)	15 (75%)
Distance education	1 (2.6%)	0 (0%)	1 (5%)
Homeschooling or unschooling	7 (17.9%)	6 (31.6%)	1 (5%)
Specialist school or support unit	5 (12.8%)	2 (10.5%)	3 (15%)
Co-occurring conditions			
ADHD	24 (61.5%)	11 (57.9%)	13 (65%)
Anxiety	23 (59%)	13 (68.4%)	10 (50%)

*Note.* NA = not applicable.

a Data are *M* (*SD*; range) or *n* (%). Percentages may not sum to 100% due to rounding issues.

### Procedure

Macquarie University’s Human Research Ethics Committee gave ethical approval for this broader study (ID 11445), which focused on the experiences of Autistic and non-autistic parents of Autistic and non-autistic children. All participants provided informed, written consent before participating.

After co-developing a semi-structured interview with an advisory group, M.H. conducted all interviews virtually. Participants could choose a synchronous (Zoom or telephone) or an asynchronous (written) interview. Only one participant in the current subset completed their interview in writing. The interview questions asked parents to describe their parenting experiences, both positive and negative, and their relationship with their child. While parents were asked about parenting challenges, no questions explicitly asked parents about child externalising or harmful behaviours. After the interview, participants provided further detailed demographic information, via REDCap (see Heyworth, 2024).

All spoken interviews were recorded and subsequently transcribed and deidentified. All participants were offered the opportunity to review their transcripts and make any edits or amendments. Participants received an AUD50 gift card for their participation.

### Data Analysis

Led by M.H., analysis was undertaken using Braun and Clarke’s (2019) six-stage process for reflexive thematic analysis, utilising an inductive approach. We adopted an interpretivist ontology, which eschews positivistic data interpretation and emphasises the role of the researcher in the language/experience nexus of meaning-making. Coding was undertaken using a semantic approach, grounded in the explicit language of participants’ experiences. Themes and subthemes were developed interpretively, where we constructed patterns between codes and explanations for these patterns to create a coherent narrative.

Although the research question focused on the moments in the interviews that pertained to a child’s harmful behaviours, analysis occurred within the context of the full interview, which often provided more context for such behaviours and parents’ responses. Codes were therefore applied to the full interviews. Initially, parent interviews were coded according to parent neurotype (Autistic and non-autistic). However, given the considerable similarities in codes – and, eventually, themes – across parent neurotypes, we combined the groups into a single dataset for this analysis. Participant quotations that reflected broader patterns within the dataset were used for theme and subtheme names to bring focus to lived and authentic experiences.

M.H. frequently conferred with D.W.T. and E.P. across all stages of the analysis. Collaboration between authors embraced researcher subjectivity, and the first author regularly shared her reflexive journey with D.W.T. and E.P. since M.H. engaged in analysis as an “insider researcher” (Braun & Clarke, 2022). As well as capturing her thoughts and reactions in a reflexive journal, M.H. openly discussed the ways that her own experiences and positioning shaped the analysis with D.W.T. and E.P. to ensure that analysis was both iterative and reflexive.

### Positionality Statement

M.H. is an Autistic researcher, mother of Autistic children, and a systemic advocate. Our research team comprises Autistic and non-autistic researchers with a deep ideological commitment to the neurodiversity paradigm and to the flourishing of the Autistic community. We are acutely aware of the harmful narratives that pervade academic and societal discourse around parenting and autism. Our purpose here is not to cast blame on parents or their Autistic children, but to illuminate the complexity of parents’ and children’s experiences to advocate for better, more knowledgeable, respectful and responsive care and support for families living in crisis and experiencing acute distress.

### Participatory Methods Statement

The broader study was informed by the guidance and experiential expertise of a paid advisory group, consisting of both Autistic and non-autistic parents of Autistic children with diverse support and communication needs; they were not participants in the study. The advisory group contributed to the study design, the interview schedule, and elements of analysis. Four participants (two Autistic and two non-autistic) from the subset described here were invited to review this manuscript prior to submission, for which they were paid additionally for their time and expertise. For a more detailed overview and reflection of the participatory process, please see Supplementary Materials S2 (Staniszewska et al., 2017).

## Results

We developed four themes related to parents’ experiences of their children’s harmful behaviours (Figure 1). Broadly, parents not only identified experiencing crises during episodes of harmful behaviour, but also actively sought to account for their child’s behaviours in ways that respected the child, separated the child from the behaviour, and prioritised connection with their child. They nevertheless reflected on the challenge of responding to such behaviours safely, and the detrimental mental impact of harmful behaviours on their own and their families’ well-being. Below, we present this pattern as themes (bold) and subthemes (italics), with illustrative quotations. Quotations are anonymised using participant IDs unique to this analysis. Non-autistic participants are indicated by the prefix “NA,” and Autistic participants by “AU.”

**Figure 1.**
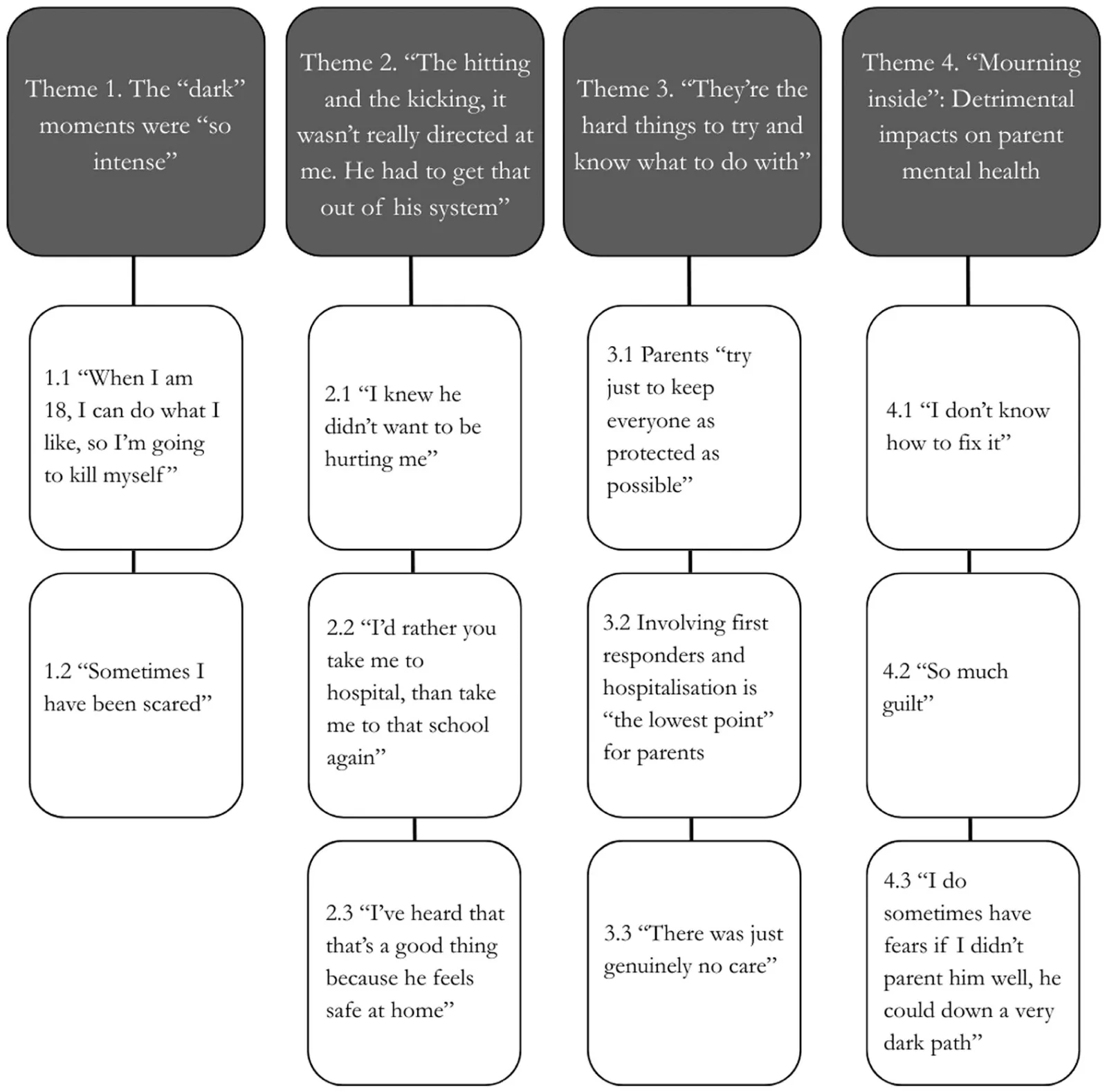
Parents’ experiences of their child’s harmful behaviours, themes, and subthemes.

Readers are advised that some material may be distressing and includes discussions of suicidal thoughts and behaviours.

### Theme 1. The “Dark” Moments Are “So Intense”

Parents described that when their child was in “some dark places” (NA1), they could become physically violent towards themselves, their parents, their siblings, others and property.

#### Subtheme 1.1. “When I am 18, I Can Do What I Like, So I’m Going to Kill Myself.”

It was “absolutely heartbreaking” (AU1) for parents when their children experienced such low mood that suicide and self-harm was a frequent danger. Children “struggle[ed] with self-harm . . . hitting himself in the head or hit[ting] his head against the wall” (AU1). Parents spoke of their child having “these quite dark thoughts from since she was little, [and] I just sometimes get this feeling like, she might be one of those people that life is just too hard” (AU2). The spectre of suicide was a constant reality for these families:

She turns to me and says, “Mummy, I can’t live like this, it’s too hard,” where I am absolutely gutted. And feel that I don’t even know what to do or say. I just wrap her up in my arms and tell her that she’s going to be okay, and all the good things about her, and her life. (AU3)

Parents described their Autistic child saying, “can you please kill me, Mummy?” (NA2), explaining that “he was just talking about hating life and not wanting to be alive anymore . . . And nothing I could do could change his mind” (NA3).

#### Subtheme 1.2. “Sometimes I Have Been Scared.”

Parents spoke about their Autistic child being violent to the parent, to the child’s siblings, or to property. Sometimes such violence was characterised as being “in an abusive relationship . . . There was a time when [husband] and I we were just covered in bruises. It was really intense” (NA4). Parents described their children “hurting me, punching me in the back of the head, and I am crying and I am running away and I’ve had to lock myself in my room” (NA5) and “he’d lay into me, hitting or kicking into me” (NA6). The consequences could be significant: “He’s broken my ribs, which is just not great” (AU5). Siblings were also targets of child violence: “he would hunt down his brother at bedtimes” (AU6), and “chase [sibling] down, and try to knock him to the ground [and . . .] just lay into him” (NA7). Property could also be a target:

He absolutely trashed the house multiple times, so many windows and even doors were broken. We had to put away all the knives and scissors and all the things we loved like our guitars so that he wouldn’t destroy them in a rage. (AU6)

Parents similarly described “hours of trashing the house” (NA8) and that “we had broken windows [and] old French doors. We ended up having to cover them with wood so that she didn’t smash through the doors” (NA4).

### Theme 2. “The Hitting and the Kicking, It Wasn’t Really Directed at Me. He Had to Get That Out of His System”

Parents made sense of their children’s harmful actions by separating the child as a person from their behaviour. Learning from the mantra that “behaviour is communication” (AU7), parents often sought to understand the underlying causes of their children’s harmful acts.

#### Subtheme 2.1. “I Feel Like It’s Not Him, It’s Not His True Self Behaving That Way.”

While some parents thought of their child as “highly aggressive and violent” (NA9), for the most part, parents understood their child’s harmful behaviour as an indicator of intense or extreme distress, rather than a personality trait. This allowed parents to justify or rationalise their child’s behaviours in ways that did not “blame” the child.

Parents recognised that when their child felt that they “have no worth” (NA2), this might prompt harmful behaviours. Equally, parents understood that “dysregulation . . . where she can’t see beyond the pain” (AU8) could make their child’s life “unbearable, he can’t cope anymore, [and] then it’s either himself or whatever he can find around him” (NA10). Parents thus acknowledged that their child’s extreme “dysregulation” (NA11) and “fight or flight kicking in all the time” (NA2) often resulted in “reactive” behaviours (AU9), which gave parents a neutral way of framing their child’s externalising behaviours. Even when parents were the targets of their child’s harmful behaviours, they understood that “the hitting and the kicking, it wasn’t really directed at me. He had to get that out of his system” (NA12). For the most part, then, parents did not attribute blame to their child, but understood their violence as a signal of extreme distress and dysregulation.

Equally, however, the way children reacted *after* harmful episodes, with intense remorse, was evidence for parents that their child was not intrinsically violent or aggressive: “he regrets it almost immediately” (AU5). Parents described that, after displaying harmful behaviours, their child “feels the most incredible remorse and shame. And that’s when he gets really down on himself and really negative, that just breaks my heart” (NA13), or “would have horrible rages and then suffer the deepest remorse, he hated himself for it” (AU6). For parents, this was evidence of their child’s real identity and personality. Consequently, parents were able to divorce their child’s behaviour from their child’s personality or identity. Parents understood that their child was “sensitive and kind and would never hurt an ant, but then at the same time can lose control of his body” (AU10).

#### Subtheme 2.2. “I’d Rather You Take Me to Hospital, Than Take Me to That School Again.”

Parents identified several stimuli *outside* of the child that might prompt such out-of-character behaviours. These accounts reflect a neurodiversity-affirming, social model approach as parents located triggers in their child’s context rather than their Autistic identity. For example, school could cause intense distress and act as a catalyst for significant behavioural changes that jarred with parents’ sense of their child’s “true nature” (AU4).

In particular, parents recognised that school was often a trigger for child self-harm and suicidality:

We were driving to school, and [Autistic child] was self-harming in the car. And she started to bleed and I said, “What are you doing?” She said, “I’d rather you take me to hospital, than take me to that school again.” (AU7)

Another parent described,

School had him in such a place that he thought he was an awful person: “I’m a bad person, I do bad things, I’m just going run on the road and die. There’s a storm, I’m going to go outside so the lightning can hit me.” (NA13)

One parent reflected that her child became unrecognisable after starting school, going from a gentle and easy-going child to “attacking me, smashing windows, making threats to burn the school down, like at school threatening to burn the school down, attacking a child” (AU13). Another described,

When he went to primary school . . . he just started to be quite dysregulated at home and started lashing out, hurting things, hurting me, and it was just weird because it didn’t seem like him, from the happy little kid that we’d known. (NA14).

School was not the only external catalyst, however, with parents noting that medication could also be a source of behavourial changes. One parent described that her child “experienced psychosis and absolute despair on that medication. He was punching himself in the face over and over again and then crying and crying because it hurt so much” (AU6).

#### Subtheme 2.3. “I’ve Heard That That’s a Good Thing Because He Feels Safe at Home.”

Parents described that they were the (sometimes sole) target of their child’s harmful episodes:

Our relationship can be difficult, in terms of when meltdowns and the physical aggression is directed purely at me . . . and especially when he bites me, and like scratching and kicking and hitting and punching. It’s quite difficult. And it’s just not the same amount of aggression towards others as it is me. (AU11)

Yet, parents justified this targeting as something positive, as a marker of being “her safe person” (AU12): “if she’s angry, upset, confused, anxious, tired, grumpy, if she’s feeling like she has no autonomy or control or power in her life, I cop the brunt of that” (AU12). This realisation did not necessarily make children’s behaviour any easier “to process” (AU11), but parents understood that “I have to still be around because he still needs mum, even though he doesn’t want mum” (NA15). As one parent put it,

He has a lot of big emotions that often result in him lashing out, and so . . . when he does lash out towards people who he feels safe with . . . I feel that he must feel some sense of safety and you know, connection in that I experience the bulk of his explosions. (NA16)

Nevertheless, by reframing their child’s harmful behaviours as an indicator of safety, parents actively attempted to see beyond the behaviour and seek connection with their child.

### Theme 3. “They’re the Hard Things to Try And Know What to Do With”

Despite parents’ sympathetic interpretations of their children’s behaviours, parents did not necessarily know how to address harmful behaviours, and they spoke at length about the need to prioritise safety and a pervasive lack of support. In other words, accounting for a child’s behaviour did not furnish parents with adequate strategies to address or circumvent such behaviours.

#### Subtheme 3.1. Parents “Try Just to Keep Everyone as Protected as Possible.”

Safety was paramount during harmful episodes, and parents had clear plans to keep their Autistic child, their family, and their property safe: “we ended up putting her in the backyard where the doors were safe, and she could be completely free to do whatever she was going to do and not come in and not hurt herself” (NA4). The safety of siblings also often preoccupied parents:

If [sibling]’s there, I have to obviously check again, safety. Who’s safe, who’s not, so there’s a plan: [sibling] might go downstairs, [sibling] might go into the room, [sibling] might go for a walk. Or go to a safe space, like a neighbour. Because you’ve got to make sure that, again, you’re ticking all the boxes for both children. (NA15)

Nevertheless, parents also recognised that what they felt was necessary to preserve safety could be distressing to them and to their child. One parent needed to leave her child to self-harm because it was the only avenue to de-escalation, but admitted that this response “is sickening, like I just want to help him. I want him to stop” (NA10). Parent and child distress was particularly high when parents felt they had no other options than to physically restrain their child: “sometimes you’ve got to pin her because she’s going after her brother . . . sometimes you’ve got no choice but to hold her, but she will be lashing out” (NA17). Not infrequently, parents felt that sacrificing their own safety to maintain sibling safety was their only recourse: “one of us would go into her room with her and lock the door because it wasn’t safe . . . We would sit in the room with her and cop it” (NA5). For many parents, then, while they understood the need to preserve safety, they felt unable to do so, with one parent even wishing her partner and non-autistic child “could live separately” (NA9) to keep them safe.

#### Subtheme 3.2. Involving First Responders and Hospitalisation Is “the Lowest Point.”

When parents felt they could not protect their own or their children’s safety, they had to involve first responders: “I can’t count how many times I held my phone with 000 on the screen reading to press ‘Call’. But I never did” (AU6).^2^ Some parents felt compelled to present to the Emergency Room with their child: “his mental health was really, really bad, to the point that we were told to go to emergency if we needed to” (AU16), or to call the police or ambulance services for support. As difficult as this decision was, such intervention could be helpful:

The lowest point was we had to call the police one time. And, as a Mum, that was a very hard thing to do. But I think that sent the message to [Autistic child] really clearly that this behaviour is not something we can live with. Like, it was, it was dangerous and something that we can’t continue to be like. (NA8)

For most, however, such intervention resulted in little tangible support or help:

Once we presented [at] the hospital. They told us he had separation issues with [wife] because he was good at school, but not good at home. So, the simple solution was, well, the problem must be at home if he can keep it together at school, so he must be fine. (NA3)

One parent described being “referred to CAMHS [Child and Adolescent Mental Health Service] at the hospital TWICE” [emphasis in written original], with parent-blaming characterising both interactions:

The first time they suggested we take “Parenting Classes” . . . and the second time, a year later, they suggested that [Autistic child]’s problems stemmed from an issue with [Autistic child] “not bonding with his mother” and suggested we take “bonding classes.” This was crushing to me, it gnawed at that insidious sense of inadequacy and guilt . . . we both felt failed by them. It was such a toxic, negative experience. Traumatic and insulting. (AU6)

The involvement of outside agencies could thus contribute to parent distress, increase parental shame and stigma through parent-blaming, and negatively impact parents’ mental health. Indeed, such involvement appeared to actively *undermine* parents’ efforts to rationalise and come to terms with their child’s behaviour by locating the cause of the behaviour in the parent.

#### Subtheme 3.3. “There Was Just Genuinely No Care.”

Parents often felt there was a systemic lack of support for them during crises, even when “we got to a place that I was like, we need help. We’re not doing this well. I don’t know what to do” (NA4). Despite needing help, one parent explained, “No one can help us . . . And I felt so broken. I was in this hole; my son was drowning. Nobody was helping me. I felt so alone” (AU14). Another parent recalled when police had been called by neighbours:

The police were called. They came to the house and they’re like . . . “Are you coping?,” and I’m like, “No, I’m not . . . .” “Oh, we’re gonna refer you to [the] D[epartment of] H[uman] S[ervices].” And I’m like, “please do; if they can help, please do.” Didn’t hear a word. So, it’s frustrating, especially when I’m asking for help. And I don’t receive help. Because the police essentially have an idea in their head that well, that child’s Autistic, so we can’t do anything to help. (AU15)

Another parent felt similarly: “I definitely needed and found that there was a massive hole in that crisis moment” (NA8). Despite a feeling that “everyone will say, ‘this is not acceptable, this is not safe, you need help’” (NA8), parents were unable to source appropriate help and reported feeling exhausted recognising that

there’s no magic pill or anything you can say to anyone where everyone drops what they’re doing and says step right up, and we’ll fix it for you. Because no matter what happens, it’s down to me and [wife] at those critical moments. (NA3)

This lack of support not only undermined parents’ attempts to manage and mitigate their child’s harmful behaviours, but also contributed to parents’ feelings of helplessness, hopelessness, and isolation.

### Theme 4. “Mourning Inside”: Detrimental Impacts on Parent Mental Health

The detrimental impacts of their children’s harmful episodes were significant, leaving parents with a deep sense of helplessness, grief and guilt, and fear for their child’s future.

#### Subtheme 4.1. “I Don’t Know How to Fix It.”

For parents, understanding their child’s harmful behaviours as an expression of intense distress helped them to account for their child’s behaviours in a validating and respectful way. Simultaneously, however, parents’ perceived inability to address or fix the source of their child’s distress, especially when external to the family (like school), left them feeling profoundly helpless. Parents admitted that being unable to help their child was “horrible and I feel so helpless” (NA3), since “I just want to make it go away for her” (AU3), and “I can’t take it all away” (NA16). In the face of her child’s suicidal ideation, one parent felt “helpless . . . and it does bring on my own depression and anxiety” (AU3). Another parent understood why her child was so distressed, but her own distress was fuelled by helplessness: “I don’t know how to fix it” (NA9), and “I feel really helpless, and I have no idea what to do” (NA18). This feeling of helplessness took its toll: “it’s just survival. It’s just doing what I’ve got to do at the time” (AU4). Parents, then, felt conflicted between the imperative to help their child and to “fix” the external circumstances triggering their child’s behaviour, on one hand, and their inability to do so effectively, on the other, which exacerbated distress and damaged parental mental health.

#### Subtheme 4.2. “So Much Guilt.”

Parents spoke about the guilt and grief they experienced reflecting on periods of crisis, described as a “sense of failure” (NA8) that “makes me really question myself as his Mum” (NA2). For some, this guilt stemmed from “my own lack of knowledge and capacity, reactivity and distress” (AU6). Another parent expressed that, in periods of crisis with her child, “there was so much going on underneath that I was unaware of. And I feel as a Mum it was my job to be aware of those. Even though I wasn’t . . . I couldn’t see those things” (NA8).

Another source of parental guilt came from their child being “still traumatised by our prior inability to see past the outbursts” (AU6) and from “[us] putting blame on him that he didn’t need as a child when . . . we didn’t understand things” (NA2). One parent explained that after an Autistic child had nearly seriously injured their sibling, she had to isolate herself from her child because “I was going to hit her so hard . . . I just could not cope [. . . so] it was like I was protecting [Autistic child] from me” (NA4). Parents thus expressed such grief and guilt as trauma, understanding that “we are all scarred from so much trauma” (AU6).

Other parents felt grief and guilt because their parental decisions had, at least in part, contributed to their child’s distress. For example, parents often felt deep guilt and grief that they had sent their children into schools which caused such damage: “I could see that I was causing that at the time. I was the one forcing him to go there” (NA19). For others again, it was the impact on siblings who “ha[ve] no choice getting exposed to the violence and the yelling” (NA18), and who “didn’t deserve any of it” (NA20) but nevertheless “suffered needlessly for years” (AU6). As one parent explained,

[Sibling]’s experiencing the same levels of yelling and violence. It’s not to him, but he sees it happening towards me, which he doesn’t cope with. He absolutely hates it when [Autistic child] yells at me and [non-autistic child] apologises all the time saying, “I’m so sorry that [Autistic child] said that to you. I’m so sorry” . . . And in my head, I’m like, well, [sibling]’s almost experiencing his own level of trauma. And that’s something that we can’t prevent him being exposed to. (NA18)

#### Subtheme 4.3. “I Do Sometimes Have Fears If I Didn’t Parent Him Well, He Could Go Down a Very Dark Path.”

Parents also spoke of fear, especially for their Autistic child’s future and of the “dark path” (NA21) that the future potentially held for their child. One parent observed,

I think parenting him into the future is really scary, extremely scary for me because he can be violent because he’s got that really quick reaction to red, to angry straightaway. There’s no thought process there and that really worries me. (NA1)

For others, that fear was more existential: “I can’t even look at school photos, if I’m being honest. You know those mug-shot photos that they have. I can’t look at them because I wonder whether this is the photo I look back on” (NA3). Parents thus feared for their child’s future and very survival, with “suicide a reality” (NA3), and an immediate and tangible fear for some families. Such fears were exacerbated by guilt, especially about the impact of “years of trauma” (AU6) on the child and their family, and helplessness from the lack of viable, effective support to help parents navigate such circumstances.

## Discussion

The parents in this study described periods of crisis when their Autistic child was exhibiting harmful behaviours, which often manifested as violence targeting self, family, and property (Theme 1). Far from blaming their children, however, parents often understood that such behaviour was a child’s (often only) way of communicating intense distress, and was not indicative of a child’s real nature, with parents recognising that their child was not inherently or innately violent (Theme 2). Parents were particularly concerned about maintaining the physical safety of all involved during harmful episodes, but this often meant they had to act in ways that were antithetical to their parenting style or values (like restraining their child), and parents felt profoundly unsupported, with limited ways to address or mitigate active crises (Theme 3). Ultimately, parents felt unable to help their children, and experienced grief, guilt, trauma, and fear (Theme 4), which had a detrimental impact on their mental health. Understanding the experiences of parents living through such crises is vital for developing responsive and respectful supports and services. This study is one step towards such understanding.

It is unsurprising that the parents in this study shared their distress at their Autistic child’s harmful behaviours (Stewart et al., 2017; Swaab et al., 2017). Yet rather than framing this distress as depression, anxiety, somatisation, or anger-hostility (Stewart et al., 2017), our participants focused specifically on the guilt they experienced because of their child’s harmful behaviours, which were often related to feelings of helplessness. While the prevalence of guilt cognitions (negative evaluation of an action; van Eickels et al., 2025) among parents of Autistic children has been established (e.g., Conrad et al., 2025; Marcinechová et al., 2024; Ostfeld-Etzion & Ben-Artzi, 2025), guilt has rarely been examined within the context of children’s harmful behaviours (cf. Swaab et al., 2017).

From a psychological perspective, the parental guilt cognitions described in this study could be interpreted as both interpersonal (specifically, omnipotent responsibility guilt; Leonardi et al., 2022) and maladaptive guilt (van Eickels et al., 2025). Omnipotent responsibility guilt refers to an escalated sense of responsibility for loved ones’ well-being and happiness (Ganguly & Tarafder, 2024). In this study, such guilt often manifested as the responsibility to “fix” the triggers for children’s harmful behaviours and to provide positive home environments, especially for siblings. While these responsibilities are generally “natural” urges for all parents, our participants often internalised their perceived failures in these areas, resulting in pervasive interpersonal guilt.

Maladaptive guilt refers to a disproportionately strong or excessive guilt, which is often triggered by misplaced or exaggerated feelings of responsibility and blame (Erb et al., 2023). Taking a functionalist approach to guilt, maladaptive guilt does not serve the same positive functions as adaptive guilt (Erb et al., 2023) and is more closely aligned to shame, since both prompt feelings of worthlessness and powerlessness (Duncan & Cacciatore, 2015; Erb et al., 2023; van Eickels et al., 2025). For our parents, maladaptive guilt was often demonstrated by grief and rumination over past decisions, especially those that might have been a catalyst to children’s harmful behaviour (like sending an Autistic child to school), for which the parent took the sole burden of responsibility. For many, such maladaptation resulted in a diffuse and all-encompassing internalised guilt, divorced from specific or changeable circumstances or actions.

Both omnipotent responsibility and maladaptive guilt characterise the experiences of parents living through crises with their Autistic children. These subtypes of guilt are correlated with poor self-esteem and mental health, including rumination, anxiety, and depression (Ganguly & Tarafder, 2024; Leonardi et al., 2022). Furthermore, maladaptive and interpersonal guilt may be implicated in the development of post-traumatic stress disorder (PTSD), especially when mechanisms of self-blame or personal responsibility are involved (Pugh et al., 2015). In addition, violating personal values – for example, restraining children or leaving them to self-harm – may similarly be implicated in the formation of PTSD (Lee et al., 2001). Given these links, there is an ethical duty to acknowledge guilt as a possible mediator in parent mental health outcomes related to child externalising behaviours, especially since supports that fail to account for such guilt will necessarily have limited efficacy (Lee et al., 2001). Thus, therapies and supports designed to help parents who are navigating these kinds of crises would benefit from incorporating self-forgiveness, unconditional self-acceptance, and self-compassion exercises to mitigate and target these experiences of guilt (e.g., Crisan et al., 2023; Marcinechová et al., 2024).

Another way to respond more appropriately to parents’ mental health needs during such crises is from within a traumatic stress framework (Stewart et al., 2016, 2017). Our parents often described events that would – directly or indirectly – be considered “large-T” complex traumas, which include repeated interpersonal violence (Straussner & Calnan, 2014). Their stories, however, also revealed many examples of “small-t” or “micro-traumas” (such as violence to property), which over time, can accumulate into significant psychological damage and distress (Mlostek, 2022). Significantly, traumas impacted not only the parent, but also the child and their siblings as well, with child trauma often exacerbating parent trauma. It would therefore be beneficial for parents of Autistic children (regardless of parent neurotype) to be screened for parenting-related trauma exposure or events (Hinde et al., 2026; Xiong et al., 2022), especially when child harmful behaviours have been reported, and for families to be offered strengths-based trauma-informed care (e.g., Bedford-Rolleston & Fenech, 2026) to address the trauma needs of the entire family. This is particularly important given that the wrong supports can actively harm – rather than benefit – individuals with traumatic disorders (Greenberg et al., 2015), who need explicit and targeted trauma-responsive therapeutic services to heal (Gjerstad et al., 2024).

Unfortunately, the efforts of our parent participants were frequently undermined both by a systemic lack of support (see also Ault et al., 2021; Papoudi et al., 2021; Seymour et al., 2022), and by the presence of disrespectful supports, which worsened crises through parent-blame (see Radev et al., 2023; Swaab et al., 2023). A lack of practical, responsive support is as deeply worrying as it is unsurprising: other studies have documented a systemic lack of care and options for managing child harmful behaviours (Holt, 2023; Swaab et al., 2023). Yet, arguably more worrying is that when parents sought help, the support they received *contributed* to their experiences of trauma and self-blame. Indeed, some parents were exposed to the antithesis of trauma-responsive care; this “support” ultimately deepened and extended periods of crisis. If parents are to provide adequate and ongoing support to their families and Autistic children, it is imperative they are supported by systems that understand their experiences and respond to them in respectful and knowledgeable ways (e.g., McCafferty & McCutcheon, 2020).

Importantly, the parents in this study offer informative ways of conceptualising Autistic children’s harmful behaviours in ways that are respectful and neurodiversity-affirming. Here, parents found ways to account for their child’s behaviour that located the stimulus for their child’s behaviours *outside* of the child, where a child’s behaviours were understood as driven by contact with substandard or harmful social and environmental contexts. Unsurprisingly, given the generally negative experiences of Autistic children in schools (e.g., Gray et al., 2023), the parents here particularly identified schools as a catalyst for their child’s harmful behaviours. However, parents understood that fixing educational – or other triggering – environments, rather than “fixing” the child, was of paramount concern when addressing child harmful behaviours. Such an affirming conceptualisation of harmful behaviours may be instructive for framing future research into, and support for, such behaviours.

### Limitations

This study has several limitations. First, while we spoke to many parents for a qualitative study, the majority identified as women, White, and well-educated, and all Autistic parents were late-identified. Moreover, the experiences of parents of children who did not use speech, or who had high communication and support needs, were underrepresented. The sample does not, therefore, reflect the full diversity of the Autistic and Autism communities. Similarly, our study was limited to parents of children in middle childhood: parents of both younger and older children may have distinct experiences not reflected by this sample. Second, as this study was not designed to examine directly parents’ experiences of Autistic children’s harmful behaviours only a subset of transcripts were analysed and no interview questions specifically probed this topic. In addition, the parents in this analysis spoke at varying length and depth about the topic. Nevertheless, a considerable amount of material spontaneously emerged on this topic, reflecting its prominence. Researchers should seek directly to examine parents’ experiences during times of crisis. Finally, some examples of harmful behaviour were excluded from this analysis (e.g., when the behaviour was nonphysical, such as verbal abuse). Given the likelihood that families will likely experience similar trauma and guilt from *non-physical* harmful behaviours, this is a limitation that future research should address.

## Conclusion

In the existing literature, child externalising behaviour has been repeatedly linked to poor parental well-being. But the stories shared by the parents in this study illustrate how complex the relationship is between children’s harmful behaviours and parental well-being. Indeed, for our participants, interpersonal and maladaptive guilt, as well as both complex- and micro-traumas, were undoubtedly factors in their mental health during and after periods of crisis. A systemic lack of support and the presence of damaging supports further exacerbated parents’ mental health outcomes. As our parents recognised, they are at the “frontline” of managing their Autistic children’s harmful behaviours, which they can only do with adequate support, for their child, and for themselves personally. In focusing on the in-depth experiences of parents themselves, we grasp the opportunity to understand ways of effectively supporting families – during times of crisis and beyond.

## Supplemental Material

sj-docx-1-aut-10.1177_13623613261476379 – Supplemental material for The Impact of “Harmful Behaviours” on Parents of Autistic Children Supplemental material, sj-docx-1-aut-10.1177_13623613261476379 for The Impact of “Harmful Behaviours” on Parents of Autistic Children by Melanie Heyworth, Diana Weiting Tan and Elizabeth Pellicano in Autism

## Data Availability

Raw qualitative data cannot be made open access due to ethical restrictions. De-identified qualitative data are available from the corresponding author upon reasonable request.

## Notes

1. We have chosen to capitalise the word Autistic to indicate a proper adjective, which reflects its status as a robust and valued identity and shared community, similar to the Deaf community.

2. In Australia, “000” is the number to call for emergency services, including police, ambulance and fire services (equivalent to “911” in the United States, “999” in the United Kingdom, or “112” in Europe).
