# Supplementary material for: The Impact of “Harmful Behaviours” on Parents of Autistic Children
Source: Autism. 2026 Aug 19;30(10):2648–61. doi: 10.1177/13623613261476379 (PMC13624354; doi:10.1177/13623613261476379)
Supplement: Supplementary file 1 [file sj-docx-1-aut-10.1177_13623613261476379.docx]

**Supplementary Materials S1. Interview schedule**

**Demographics**

What is your date of birth (DOB)?

What is your postcode?

Are you formally diagnosed as Autistic, self-identifying, or questioning?

At what age did you receive your Autism diagnosis?

What is your Autistic child’s first name?

What is their date of birth?

What is your child’s diagnosis? When did they receive it?

*[When applicable]*

What is your non-autistic child’s first name?

What is their DOB?

**Speech Sample**

Firstly, I’d like to hear your thoughts about [Autistic child] in your own words and without my interrupting you with any questions or comments. When I ask you to begin, I’d like you to speak for 5 minutes. When you’ve spoken for 5 minutes, I’ll let you know. After you have begun to speak, I prefer not to answer any questions. So, I’d like you to tell me what kind of a person [Autistic child] is and how the two of you get along together. Are there any questions you would like to ask me before we begin?

*[When applicable]*

Now, I’m going to ask the same question about [non-autistic child]. I’d like to hear your thoughts about [non-autistic child] in your own words and without my interrupting you with any questions or comments. When I ask you to begin, I’d like you to speak for 5 minutes. When you’ve spoken for 5 minutes, I’ll let you know. After you have begun to speak, I prefer not to answer any questions. So, I’d like you to tell me what kind of a person [non-autistic child] is and how the two of you get along together. Are there any questions you would like to ask me before we begin?

*[Written version]*

Firstly, I’d like to hear your thoughts about [Autistic child] in your own words. This is meant to be a spontaneous response, so it doesn’t need to be polished or edited in any way. When you begin writing, I’d like you to write around 650-750 words. When a participant responds to this question in speech, I try not to answer any questions or interrupt them, so I would love you to try and write your answer just to this question in one sitting, and without further clarification from me if that is feasible.

So, I’d like you to tell me what kind of a person [Autistic child] is and how the two of you get along together.

**Semi-structured interview**

1a) Tell me more about your experiences of parenting [Autistic child].

Prompts:

- What are some of the fulfilling or joyful parts of parenting [Autistic child]?
- What are some of the challenging parts of parenting [Autistic child]?
- What are some of your day-to-day or week-to-week routines that seem to shape the rhythm of your time as a parent?
- What sorts of things do you do with [Autistic child]?

*[When applicable]*

1b) Are there differences in your experiences in parenting [Autistic child] and [non-autistic child]?

Prompts:

- If you reflected on your experiences of parenting [Autistic child] and [non-autistic child], are the parts that are fulfilling or challenging different? If so, how?
- Are the routines you have with [non-autistic child] different to those you have established with [Autistic child]? What is the effect or impact of those differences?
- What sorts of things do you do with [non-autistic child]? Are these different to [Autistic child]? If yes, why? What is the effect or impact of those differences?

2a) How would you describe your attachment – or your connection – with [Autistic child]?

Prompts:

- Are there moments where you feel a deep connection with [Autistic child]? What are they?
- Are there times when you feel disconnected from [Autistic child]? Tell me about them.
- What emotions do you feel about [Autistic child] or how do they make you feel?
- Do you think [Autistic child] reciprocates these feelings? What indicates that?

*[When applicable]*

2b) Any there any differences in your feelings of attachment and connection with [non-autistic child]?

Prompts:

- Are there moments where you feel a deep connection with [non-autistic child]? What are they?
- Are there times when you feel disconnected from [non-autistic child]? Tell me about them.
- What emotions do you feel about [non-autistic child] or how do they make you feel?
- Do you think [non-autistic child] reciprocates these feelings? What indicates that?

2c) What is the thing that, in your opinion, most characterises the relationship between you and [Autistic child]?

What are your expectations for the future regarding your relationship with [Autistic child]?

*[When applicable]*

2d) What is the thing that, in your opinion, most characterises the relationship between you and [non-autistic child]?

What are your expectations for the future regarding your relationship with [non-autistic child]?

3) Tell me about how good a job you feel you are doing as a parent.

Prompts:

- Do you think you do a good job parenting? What areas do you think you do really well in?
- Are there times you don’t think you’re a very good parent? Tell me about them.
- Do you think you do as good a job parenting your different children? What elements play into how well you think you’re doing?

4) Tell me a bit about when your child experiences “big emotions”; how do you respond as a parent?

Prompts:

- *[When applicable]* Are your parenting responses the same for all your children? Why/why not?
- What factors play into your response to your child/ren’s “big emotions”?
- What do you think about your child/ren when they are experiencing “big emotions”? *[When applicable]* Is this different for your different children?

5) Do you feel you understand why your child/ren behave the way they do?

Prompts:

- Do you think you understand the motivations underlying your child/ren’s behaviours?
- *[When applicable]* Is this the same for all your children?
- What helps you to understand your child/ren’s motivations?

6) What are your past experiences of therapies and interventions?

Prompts:

- Did any past therapies or interventions focus on your parenting? How did that make you feel? Do you think they worked?
- How do you think these have influenced your relationship with your child?

7) Remembering the time your Autistic child was diagnosed Autistic, what information or support do you wish you had been given then, to help you in your parenting journey?

Is there anything else you would like to add, or want me to know, that you haven’t had the opportunity to talk about?

**End of formal questions.**

**Supplementary Materials S2. Authors’ review of their participatory approach based on Staniszewska et al.’s (2017) Guidance for Reporting Involvement of Patients and the Public Version 2 (Short Form) (GRIPP-2)**

| **1. Aim**  Report the aim of the study | The aim of this study was to understand the experiences of parents of Autistic children who displayed harmful behaviours (defined as violence to self, others and property). Specifically, we examined the nature of these behaviours, parents’ accounts and responses, and self-reported impact of these behaviours on parents.  The involvement of an Autistic researcher ([anonymised for review]), a community advisory group of parents of Autistic children, and additional member checking was required to ensure that:   1) The “complex emotional terrain” (Holt, 2024, p. 814) examined in this analysis was captured in ways that were respectful of both parents and children, and with appropriate nuance and regard for the research inheritance of “parent blaming” 2) The Autistic community felt that the manuscript contributed in constructive ways to research fostering flourishing. |
| --- | --- |
| **2. Methods**  Provide a clear description of the methods used for community involvement in the study | The study was first conceptualised by [anonymised for review], an Autistic researcher, parent to Autistic children, and systemic advocate for the Autistic community.  [Anonymised for review] formed an advisory group of both Autistic and non-autistic parents of Autistic children with diverse support and communication needs. This advisory group was involved in study meetings and invited to review material relating to the project, including: study design, study materials (recruitment, information and consent protocols, interview schedule, background questionnaire), analysis, knowledge translation, and manuscript review. They were paid for their time.  Prior to submission, four parents (two Autistic and two non-Autistic) from the subset of participants used for this analysis reviewed the final manuscript to ensure it was respectful of and sensitive to their and their children’s experiences. Potential reviewers were warned in advance of the contents of the manuscript to avoid possible re-traumatisation.  They were paid for their time. |
| **3. Results**  Outcomes – Report the results of community involvement in the study, including both positive and negative outcomes | Beyond [anonymised for review], who led the project, the advisory group and reviewers contributed to this analysis by:   - Contributing to the discussion about the role and impact of guilt on parents’ mental health (e.g., resonating with the importance of guilt even outside of harmful behaviours) - Interpreting results (e.g., the connection between guilt, values, decision-making, and lack of support; the difference between adaptive guilt and shame or maladaptive guilt; the importance of considering sibling dynamics) - Providing feedback on drafts of manuscript |
| **4. Discussion**  Outcomes – Comment on the extent to which PPI influenced the study overall. Describe both positive and negative effects. | Whilst [anonymised for review] is Autistic with lived experience of parenting Autistic children, she is nevertheless a researcher. The advisory group and reviewers were thus vital to ensure representation from the lay community, to ensure that experiential knowledge was respectfully captured and valued alongside research knowledge. Given the difficult topic and the potential for readers to interpret parent-blame and/or child-/autism-blame into the findings, it was thus deemed imperative to check that parents felt that their complex experiences were validated and respected and that they agreed that the analysis did not apportion blame to them or their children.  The parents who reviewed this manuscript felt it “represents our experiences with such truth, depth and accurate detail” and that it was “very precious and meaningful for me” since “I can’t really express how important I think this paper is”. Moreover, these parents felt that “these kinds of studies can help kick the cogs of change into gear [so that] families of the future do not have to suffer as intensely as those of us who participated here” and that “it should be compulsory reading for MH staff at hospitals, emergency services, educators”. They also felt that the study created “a space where our stories were honoured with such care”.  Despite being paid for their time to review the manuscript prior to publication, we recognise that being asked to review may have put undue burden on participants. As much as we made every effort to represent experiences respectfully, the possibility that reading their own and others’ similar experiences captured would re-traumatise participants is undeniable. We were thus caught between wanting to ensure that the paper was acceptable to participants, whilst not wanting to expose participants to potentially triggering content. Ideally, with sufficient time and funding, we would have asked *all* parents included in this analysis to comment on the manuscript, but we did not have the resources for that process for this paper. |
| **5. Reflections**  Critical perspective – Comment critically on the study, reflecting on the things that went well and those that did not, so others can learn from this experience. | Whilst having Autistic representation on the research team was important, it was equally important to have adequate lay community and experiential representation. Lay community representation ensures that research meets the needs and priorities of the community, and reorients research from a transactional process to a relational one. For example, the advisory group emphasised the need for effective knowledge translation to the target community, highlighting the importance of “giving back” to the community.  However, community representation needs to expand beyond simply, for example, Autistic involvement. Prioritising relevant experiential knowledge is vital in ensuring that the specific needs and experiences of unique and focused populations are represented in ways that are validating, respectful, nuanced, sensitive, and – ultimately – helpful. That is, through experiential input we came closer to presenting difficult moments in ways that resonated and were cathartic and hopeful, rather than simply traumatising. |
